# Chronic low dose of AM404 ameliorates the cognitive impairment and pathological features in hyperglycemic 3xTg-AD mice

**DOI:** 10.1007/s00213-018-5108-0

**Published:** 2018-11-13

**Authors:** Hei-Jen Huang, Shu-Ling Chen, Hsin-Yu Huang, Ying-Chieh Sun, Guan-Chiun Lee, Guey-Jen Lee-Chen, Hsiu Mei Hsieh-Li, Ming-Tsan Su

**Affiliations:** 1 Department of Nursing, Mackay Junior College of Medicine, Nursing and Management, Taipei, 11260 Taiwan; 2 Department of Life Science, National Taiwan Normal University, Taipei, 11677 Taiwan; 3 Department of Chemistry, National Taiwan Normal University, Taipei, 11677 Taiwan

**Keywords:** Hyperglycemic, Alzheimer’s disease, AM404, Therapeutics

## Abstract

**Rationale:**

Hyperglycemia accelerates the progression of Alzheimer’s disease (AD), and GSK3β plays a potential link between AD and hyperglycemia. Therefore, a direct or indirect GSK3β inhibition may have potential to delay the progression of AD. Our previous biochemical assay identified AM404 as a GSK3β inhibitor at high dose (IC50 = 5.353 μM); however, other study suggests that AM404 impaired synaptic plasticity of hippocampus at high dose (10 mg/kg; i.p.). Therefore, the dose and duration of treatment are crucial for the effects of AM404.

**Objective:**

The effects and molecular mechanisms of AM404 at low dose were evaluated from in vitro to in vivo models.

**Methods:**

AM404 (0.1–0.5 μM) was tested on tau hyperphosphorylated mouse hippocampal primary cultures treated with Wortmannin (WT) and GF109203X (GFX). Hyperglycemic triple transgenic AD (3×Tg-AD) mice at 6 months old were intraperitoneally inject*e*d with AM404 (0.25 mg/kg) for 4 weeks. The spatial learning and memory of mice were measured using the Morris water maze. Mouse brain and serum samples were collected for pathological analyses.

**Results:**

AM404 (0.5 μM) exhibited significantly augmented neuroprotection toward tau hyperphosphorylation in primary cultures. The chronic systemic administration of AM404 (0.25 mg/kg) attenuated cognitive deficits in hyperglycemic 3×Tg-AD mice. Moreover, chronic low dose of AM404 significantly attenuated Aβ production, tau protein phosphorylation, and inflammation associated with an increase of pS473Akt and pS9-GSK3β. Therefore, AM404 at low dose, not only increased neuroprotection, but also ameliorated cognitive deficit, could be partly by regulating the Akt/GSK3β signaling, which may contribute to downregulation of Aβ, tau hyperphosphorylation, and inflammation in hyperglycemic 3×Tg-AD mice.

**Conclusions:**

These results highlight that chronic administration of AM404 at low dose may be through the Akt/GSK3β pathway to ameliorate the impairment in hyperglycemic 3×Tg-AD mice.

**Electronic supplementary material:**

The online version of this article (10.1007/s00213-018-5108-0) contains supplementary material, which is available to authorized users.

## Introduction

Alzheimer’s disease (AD) is the most common progressive neurodegenerative disease. The annual incidence of AD is currently estimated at 4.6 million cases, and as the general population ages, the worldwide prevalence is expected to increase to more than 80 million cases by the year 2040 (Ferri et al. 2005). Currently, the treatment options for AD only ameliorate the symptoms and do not inhibit the natural progression of AD. Strong evidence has shown that amyloid beta (Aβ) plaques and tau-associated neurofibrillary tangles (NFTs) “clog” the brain and prevent neuronal communication in patients with AD, causing further brain dysfunction (Braak and Braak 1991a, b). Many studies have found that both Aβ and tau drive the pathogenesis of AD by modulating glycogen synthase kinase 3 (GSK3) activity (Querfurth and LaFerla 2010). Accumulating evidence has demonstrated that GSK3β plays a role in tau hyperphosphorylation and the associated memory impairment in AD (Dolan and Johnson 2010). In addition, the oxidative stress, inflammation, and neuroinflammation associated with AD are also mediated by GSK3β activation (Zhang et al. 2015; D’Angelo et al. 2016). Evidence also shows that GSK-3β is a potential link between diabetes mellitus (DM) and AD (Zhang et al. 2018), and DM might accelerate the progression of AD (Guo et al. 2016). According to the announcement by Jackson laboratory at February 2014, male 3xTg-AD mice may not exhibit the phenotypic traits originally described by donating investigator. Therefore, hyperglycemia was applied to accelerate the phenotypes of 3xTg-AD mice at 6 months old in the study.

Extensive evidence has shown that the endocannabinoid system is an important regulator of synaptic function (Hill et al. 2010; Campolongo et al. 2011). Animal studies have also demonstrated that the endocannabinoid system modulates recognition memory by selectively affecting encoding within the hippocampus (Barna et al. 2007). *N*-arachidonoylphenolamine (AM404), a paracetamol lipid metabolite, acts as a modulator of the endocannabinoid system and is endowed with pleiotropic activities including activating transient receptor potential vanilloid receptor 1 (TRPV1), inhibiting fatty acid amide hydrolase (FAAH)-mediated hydrolysis of *N*-arachidonoyl ethanolamide (anandamide; AEA), and inhibiting the synthesis of cyclooxygenase (COX)-1, COX-2, and prostaglandin (Hogestatt et al. 2005). Several studies have also shown that AM404 has a role in the extinction of fear memories in rats (Bitencourt et al. 2008), produces anxiolytic and antidepressant-like effects in rats and mice (Patel and Hillard 2006; Adamczyk et al. 2008), and is neuroprotective in ischemic gerbils (Zani et al. 2007) and rats with Parkinson’s disease (Garcia-Arencibia et al. 2007). We previously utilized a virtual screening system and a series of biochemical assays to show that the concentration of AM404 necessary to half-maximally inhibit GSK-3β kinase activity (IC50) is 5.353 μM (Lin et al. 2016). Using cell-based assays, we also showed that AM404 increased Ser 9-phospho-GSK3β (pS9-GSK3β) expression, reduced tau phosphorylation, enhanced heat shock 27 kDa protein 1 (HSPB1) and glucose-regulated protein 78 kDa (GRP78) expression, increased tau solubility, and promoted neurite outgrowth (Lin et al. 2016). However, previous studies suggests that intraperitoneal injection of AM404 (10 mg/kg) not only impaired long-term potentiation through the hippocampal Schaffer collateral-CA1 projection (Abush and Akirav 2010) but also potentiated restraint-induced corticosterone release (Patel et al. 2004). Evidence from clinical trial further points out that the effects of GSK3β inhibitors were correlated to the dose and duration of treatment in AD (Lovestone et al. 2015). Based on these reports, the current study was designed to examine whether AM404 at low dose exerts neuroprotective effects and improves cognitive deficits and pathological features of AD in primary murine hippocampal neuron cultures and hyperglycemic 3×Tg-AD mice.

In this study, AM404 at 0.5 μM exerted neuroprotective effects on primary murine hippocampal neuron cultures by inhibiting tau toxicity. In addition, chronic administration of AM404 at low dose (0.25 mg/kg; i.p.) improved spatial cognition and attenuated pathological features, which might be in part by the Akt/GSK3β pathway in hyperglycemic 3×Tg-AD mice. Therefore, we suggest that chronic systemic administration of AM404 at low dose could be a potential therapeutic for AD, which might be through Akt/GSK3β pathway, not dependent by a direct GSK3β inhibitor, or receptor of CB1 and TRVP1.

## Materials and methods

### Animals

Pregnant female C57BL/6J and male 3×Tg-AD mice were purchased from the National Breeding Centre for Laboratory Animals (Taipei, Taiwan) and Jackson Laboratory (004807; Bar Harbor, ME, USA), respectively. Mice were housed in individual ventilated cages at 20–25 °C, with free access to food and water and a 12/12-h light/dark cycle (7 AM to 7 PM). Mice were deeply anesthetized with Avertin (0.4 g/kg body weight, Sigma, St. Louis, MO, USA) and then sacrificed to conduct pathological analyses; all efforts were made to minimize suffering.

### Experimental timeline for the 3×Tg-AD mice

Male 3×Tg-AD mice obtained from Jackson Laboratory (004807; Bar Harbor, ME, USA) only exhibit a mild phenotype at 6 months of age. Therefore, hyperglycemia was induced using streptozotocin (STZ) to accelerate disease progression in the 6-month-old male 3×Tg-AD mice. Following a 6-h fasting period, STZ (100 mg/kg in 0.1 ml sodium citrate buffer, pH 4.5, Sigma, St. Louis, MO, USA) was intraperitoneally (i.p.) injected into the 3×Tg-AD mice on days 1, 2, 8, and 9 to induce hyperglycemia (*n* = 30). An equivalent volume of sodium citrate buffer was i.p. injected into a subgroup of mice (*n* = 30) to create a normoglycemic group (NBG). Blood samples were obtained from all mice by tail prick, and glucose levels were measured using a commercial glucometer (Accu-Check Active, Roche, Mannheim, Germany). The STZ-treated mice that had developed hyperglycemia by day 14 (defined as a blood glucose concentration ≥ 200 mg/dl) were included in the study as the hyperglycemic group (HBG). The dose of AM404 (0.25 mg/kg/day; Sigma) was determined based on the results obtained from primary hippocampal neuronal cultures (Supporting Information S1 and S2) and was slightly modified according to the dose used in a previous study (Miller et al. 2014). AM404 was i.p. injected into the mice starting on day 14 and lasting for 4 weeks. The mice were divided into four groups: (i) NBG/vehicle (*n* = 15), (ii) NBG/AM404 (*n* = 15), (iii) HBG/vehicle (*n* = 8), and (iv) HBG/AM404 (*n* = 9). Body weights and blood glucose levels were monitored weekly during the treatment (Supporting Information S3). The Morris water maze (MWM) was conducted on days 34–42 to evaluate the spatial learning abilities and memory capacities of the mice. The mice were sacrificed on day 43 for pathological and molecular characterization (Supporting Information S3).

### MWM

The MWM was performed based on previously described methods to evaluate the spatial learning abilities and memory capacities of the treated mice (Huang et al. 2012). Briefly, a circular pool (height 47 cm, diameter 100 cm) was filled with opaque water (24 ± 1 °C), and a hidden square platform (10 cm^2^) was submerged 1 cm below the surface of the water in the center of the northeast quadrant, which served as the target quadrant. A video camera was attached to the ceiling to record the behavior of the mice in the pool, which was analyzed using EthoVision software (Noldus, Wageningen, Netherlands). During the four training days, each mouse underwent four trials per day. Each mouse was released into the water maze from a random starting point in each trial. The time that each mouse spent locating the hidden platform (i.e., the escape latency) was calculated, and a curve was generated to represent the data collected over the four training days, which represented the learning profiles of the mice. Twenty-four hours after the final training trial, three testing trials were conducted to determine the time required to find the hidden platform as a measure of spatial learning acquisition. A probe trial was conducted 48 h after the end of the testing trials to evaluate long-term spatial memory. Each mouse was allowed to swim freely for 60 s, and the time the mouse spent in the target quadrant (where the platform was removed) was measured to represent the degree of memory consolidation after learning.

### Western blot analysis

Mouse hippocampal tissues (*n* = 3–5 per group) were homogenized, and the concentrations of the isolated proteins were determined using a bicinchoninic acid (BCA) protein assay kit (Thermo Fisher Scientific, Rockford, IL, USA). The homogenates (25 μg of protein each) were subsequently separated using SDS-PAGE and transferred to polyvinylidene fluoride (PVDF) membranes. Blots were then blocked in 5% (*w*/*v*) skim milk to reduce nonspecific binding and probed with the following primary antibodies: pS473Akt (1:1000; Cell Signaling Technology, Danvers, MA, USA); Akt (for Akt1–3; 1:1000; Cell Signaling Technology, Danvers, MA, USA); pS9-GSK3β (1:1000; Cell Signaling Technology, Danvers, MA, USA); pY216-GSK3β (1:1000; Millipore, Temecula, MA, USA); GSK3β (1:1000; Cell Signaling Technology, Danvers, MA, USA); HT7 (1:500; Thermo Fisher Scientific, Rockford, IL, USA); tau phosphorylated at S396 (1:1000; Invitrogen, Rockford, USA), S202 (1:1000; AnaSpec, San Jose, CA, USA), and T231 (1:1000; Invitrogen, Rockford, IL, USA); beta-secretase 1 (BACE1; 1:1000; Cell Signaling Technology, Danvers, MA, USA); insulin-degrading enzyme (IDE; 1:1000; Abcam, Cambridge, UK); neprilysin (NEP; 1:1000; Santa Cruz Biotechnology, Dallas, Texas, USA); and β-actin (1:2000; Millipore, Temecula, CA, USA). These incubations were followed by incubations with horseradish peroxidase (HRP)-conjugated anti-mouse or anti-rabbit IgG (1:10,000; Amersham Pharmacia Biotech, Piscataway, NJ, USA). The protein bands were scanned using an enhanced chemiluminescence detection system (Amersham Pharmacia Biotech, Piscataway, NJ, USA). The immunoreactive bands were quantified using an LAS-4000 chemiluminescence detection system (Fujifilm; Tokyo, Japan).

### Immunohistochemical staining

Immunohistochemical (IHC) staining was conducted as previously described (Huang et al. 2015). Briefly, after the MWM, the mice (*n* = 3–5 per group) were deeply anesthetized with Avertin (0.4 g/kg body weight) and then perfused through the heart with normal saline followed by ice-cold 4% PFA in 0.1 mol/L phosphate-buffered saline (PBS, pH 7.0). The brains of the mice were then removed and post-fixed in the same solution at 4 °C for 24 h. Then, the brains were dehydrated in a graded series of sucrose solutions until they were fully permeated. Thereafter, 30-μm-thick frozen sections were cut in the coronal plane using a cryostat microtome (CMS3050S; Leica Microsystems; Nussloch, Germany). The free-floating sections were incubated with primary antibodies against Aβ_40_ (1:2000; Invitrogen, Rockford, IL, USA), Aβ_42_ (1:500; Invitrogen, Rockford, IL, USA), tyrosine hydroxylase (TH; for noradrenergic neurons; 1:1000; Millipore, Temecula, CA, USA), 5-hydroxytryptamine (5HT; for serotonergic neurons; 1:200; Millipore, Temecula, CA, USA), choline acetyltransferase (ChAT; for cholinergic neurons; 1:1000; Millipore, Temecula, CA, USA), Iba-1 (for microglia; 1:1000; Wako, Osaka, Japan), and glial fibrillary acidic protein (GFAP; for astrocytes; 1:1000; Millipore, Temecula, CA, USA) overnight at room temperature. On the second day, the sections were washed in PBS and incubated with the appropriate biotinylated secondary antibodies (1:200 dilution; Vector Laboratories, CA, USA) followed by incubation with an avidin–biotin–peroxidase complex (Vectastain Elite ABC kit; Vector Laboratories, CA, USA) for 1 h at room temperature. Immunoreactivity was visualized with a diaminobenzidine (DAB) kit (Vector Laboratories, CA, USA). After terminating the reaction by the addition of PBS, the sections were mounted, dehydrated, and coverslipped. For each analysis, three slices of each mouse hippocampus and brainstem were stained, and three to five mice were used for each group. For each slice, the Aβ_40_ and Aβ_42_ staining in the CA1 subregion of the hippocampus, astrocyte (GFAP) and microglia (ionized calcium-binding adapter molecule 1, Iba1) staining in the whole hippocampus, and serotonergic (5-HT) and noradrenergic neuron (TH) staining in the raphe nucleus and locus coeruleus (LC) region of the brainstem were observed using a light microscope (Leica; Wetzlar, Germany). All images were analyzed using digital image analysis software Image Pro Plus (IPP; Media Cybernetics, MD). A positive-staining cell in the specific area, such as hypertrophic astrocytes; activated microglia, Aβ_40_, and Aβ_42_ in hippocampus; serotonergic neurons in Raphe nucleus; and noradrenergic neurons in LC region was selected as a standard signal, and the numbers of positive cells were then counted by the IPP software system. Pixel counts were derived from the average of three adjacent sections per animal.

### Enzyme-linked immunosorbent assay analysis

Prior to sacrifice, blood samples were collected from the mice via a facial vein using a lancet (*n* = 3–5 per group). The blood samples were centrifuged at 2000×*g* for 20 min at 4 °C. Thereafter, the supernatants were collected as serum samples and stored at − 80 °C until further examination. The levels of interleukin-6 (IL-6) and tumor necrosis factor-alpha (TNF-α) in the serum samples were measured using enzyme-linked immunosorbent assay (ELISA) kits (R&D Systems; Minneapolis, MN, USA).

### Data analysis

All values are expressed as means ± standard errors of the means (SEM). The statistical analyses were performed using SPSS 16.0 software. The interactions and effects between the factors in the MWM were analyzed using a two-way analysis of variance (ANOVA, general linear model). The other data were analyzed using a one-way ANOVA followed by a least significant difference (LSD) post hoc test. Comparisons between two groups were performed using independent Student’s *t* tests or nonparametric tests (Kruskal–Wallis test). For all tests, *P* < 0.05 was considered significant.

## Results

### AM404 attenuates spatial learning and memory impairments in hyperglycemic 3×Tg-AD mice

**Fig. 1:**
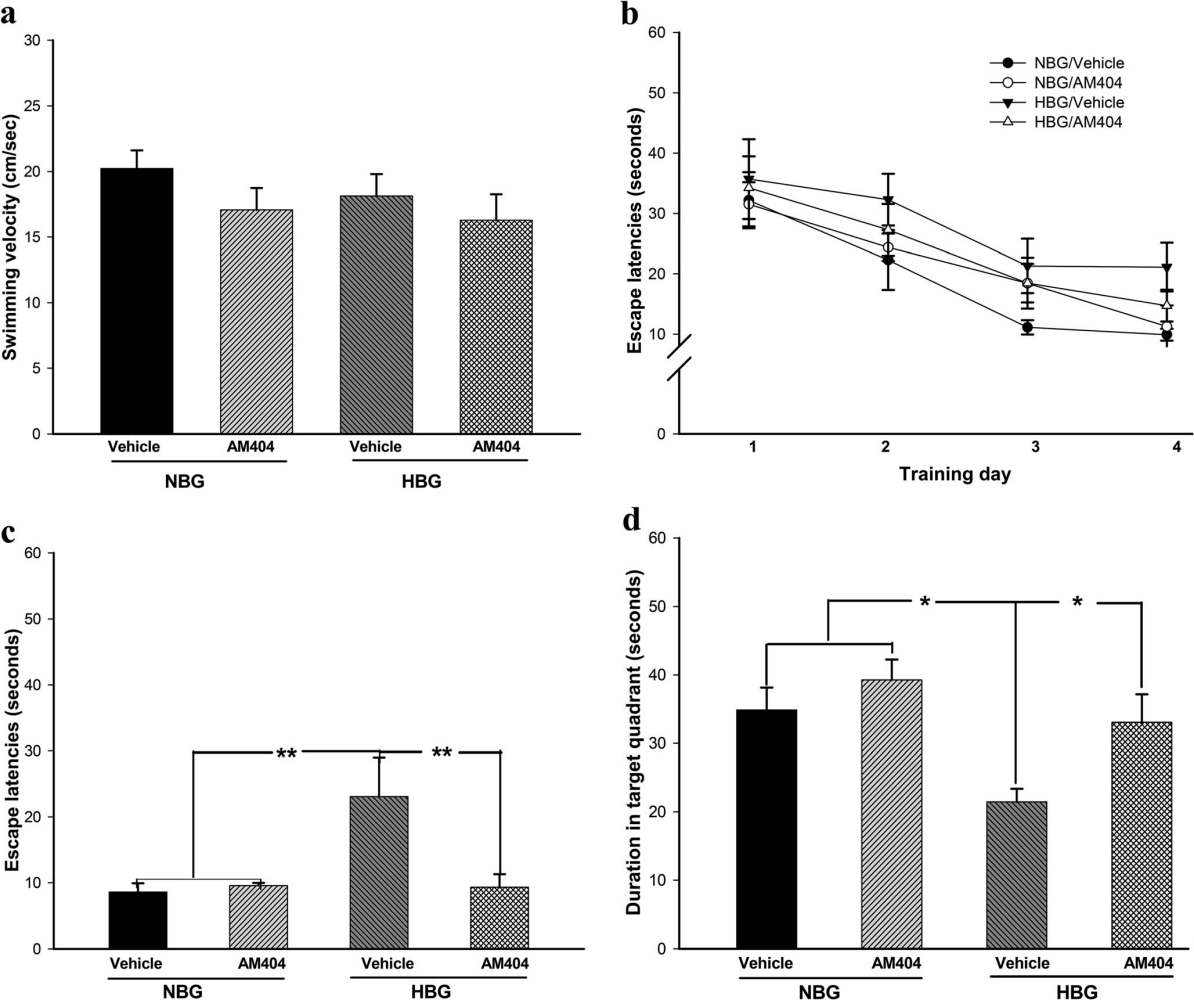
Effects of AM404 on the learning and memory capacities of hyperglycemic 3×Tg-AD mice. **a** Swimming velocity was measured on the testing day. No differences were identified among the four groups of mice. **b** The learning profiles of the four groups of mice during the four training days. AM404 improved the learning ability in the HBG groups. **c** Latency in the identification of the platform during the testing trials. The hyperglycemic mice spent more time finding the platform than the control mice, and AM404 treatment ameliorated this impairment. **d** The results of the long-term memory retrieval testing. Hyperglycemia enhanced the impairment of long-term memory retrieval, and AM404 attenuated this deficit. Data are presented as the means ± SEM of each group. *n* = 15 (NBG with vehicle group), 15 (NBG with AM404 group), 8 (HBG with vehicle group), and 9 (HBG with AM404 group). *P < 0.05; **P < 0.01

The neuroprotective effect of AM404 on tau hyperphosphorylation in primary murine hippocampal neurons and *Drosophila* model has been confirmed (Supporting Information S1–S3). Therefore, AM404 was further applied to hyperglycemic 3×Tg-AD mice to validate its in vivo function. AM404 had no effects on body weights or blood glucose levels in these mice (Supporting Information S4). The MWM was conducted on days 34–42 to evaluate the spatial learning abilities and memory capacities of the mice. Based on the results of the two-way ANOVA, no significant interactions were observed between hyperglycemia and AM404 with regard to swimming velocity, spatial learning ability, or spatial memory retrieval in the 3×Tg-AD mice (data not shown). In addition, no differences in swimming velocity were observed among the four groups of mice (Fig. 1a). During the four training days, the NBG groups presented normal learning abilities following the administration of vehicle (*F*(3,19) = 4.799, *P* < 0.05; Fig. 1b) or AM404 (*F*(3,24) = 3.801, *P* < 0.05; Fig. 1b). The vehicle-treated HBG group showed significantly poorer spatial learning abilities from training days 1 to 4 (*F*(3,24) = 2.201, *P* > 0.05; Fig. 1b). The AM404 treatment significantly reduced the spatial learning impairments in this group (*F*(3,15) = 3.782, *P* < 0.05; Fig. 1b). In addition, the STZ injections impaired the acquisition of spatial learning during the test trial (*P* < 0.01, Fig. 1c) and long-term memory retrieval during the probe trial (*P* < 0.05; Fig. 1d) in the HBG group, whereas the AM404 treatment ameliorated the impairments in spatial learning acquisition (*P* < 0.01; Fig. 1c) and memory consolidation (*P* < 0.05; Fig. 1d).

### AM404 upregulates Akt and reduces tau phosphorylation in hyperglycemic 3×Tg-AD mice

**Fig. 2:**
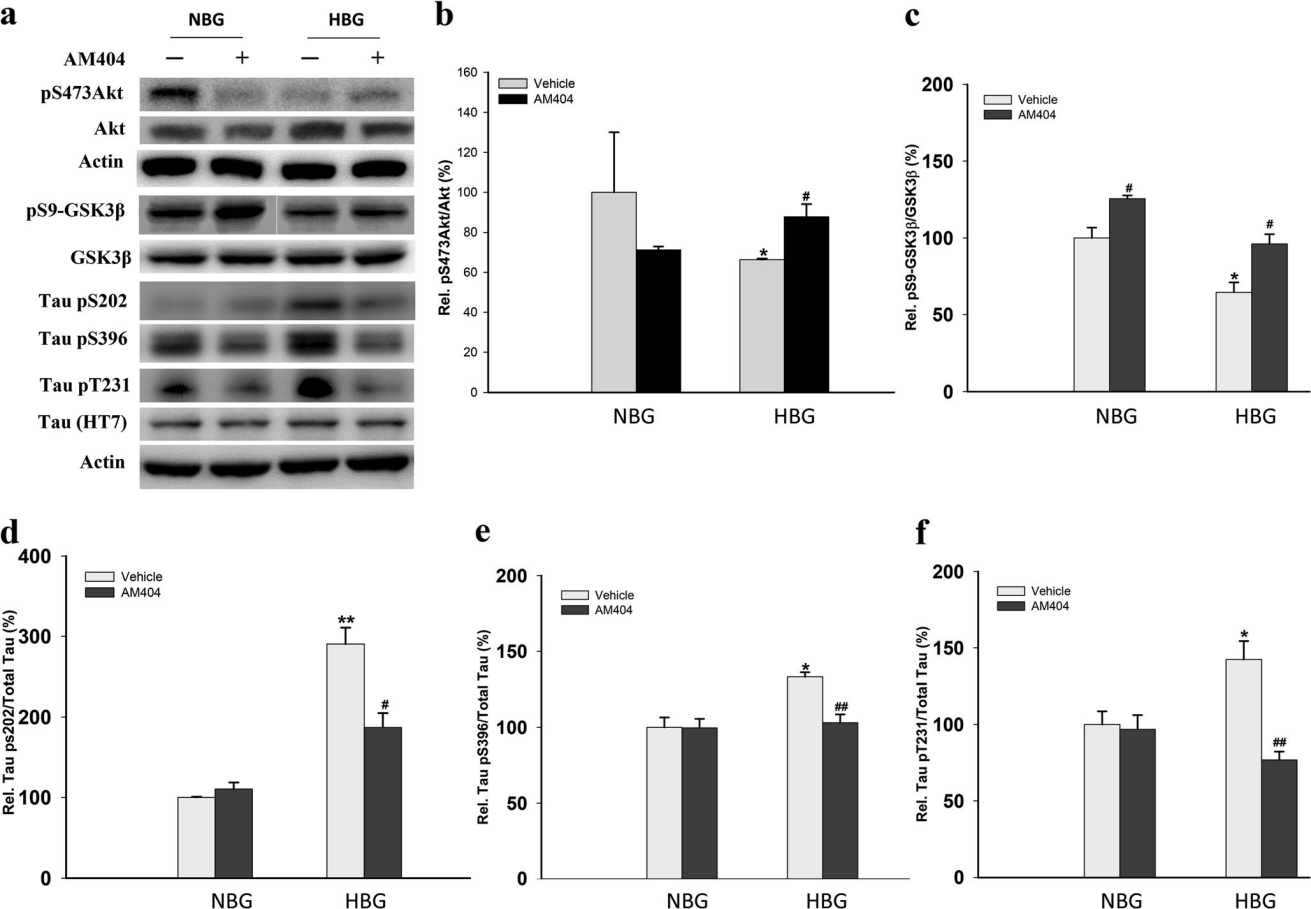
Effect of AM404 on tau hyperphosphorylation in hyperglycemic 3×Tg-AD mice. Representative western blots (**a**) and quantitative densitometry results for the pS473Akt/Akt ratio (**b**), pS9-GSK3β/GSK3β ratio (**c**), and ratio of tau phosphorylated sites Ser202 (**d**), Ser396 (**e**), and Thr231 (**f**) to actin. β-Actin was used as an internal control. The quantitative data are presented as the means ± SEM of each group (*n* = 3–5 per group). Asterisk, compared to NBG with vehicle group; number sign, compared to HBG with vehicle group (*^,#^*P* < 0.05; **^, ##^*P* < 0.01)

Mice were sacrificed after the MWM task, and their hippocampi were isolated for western blot analysis (Fig. 2a). The ratios of both pS473Akt/Akt and pS9-GSK3β/GSK3β were decreased in the HBG group (*P* < 0.05; Fig. 2b, c). The levels of the tau protein phosphorylated at Ser202, Ser396, and Thr231 were also increased in this group (*P* < 0.05–0.01; Fig. 2d–f). All of the indices related to the tau toxicity induced by hyperglycemia were attenuated by the AM404 treatment (*P* < 0.05–0.01; Fig. 2b–f) (*n* = 3–5).

### AM404 attenuates Aβ levels and BACE1 expression in hyperglycemic 3×Tg-AD mice

**Fig. 3:**
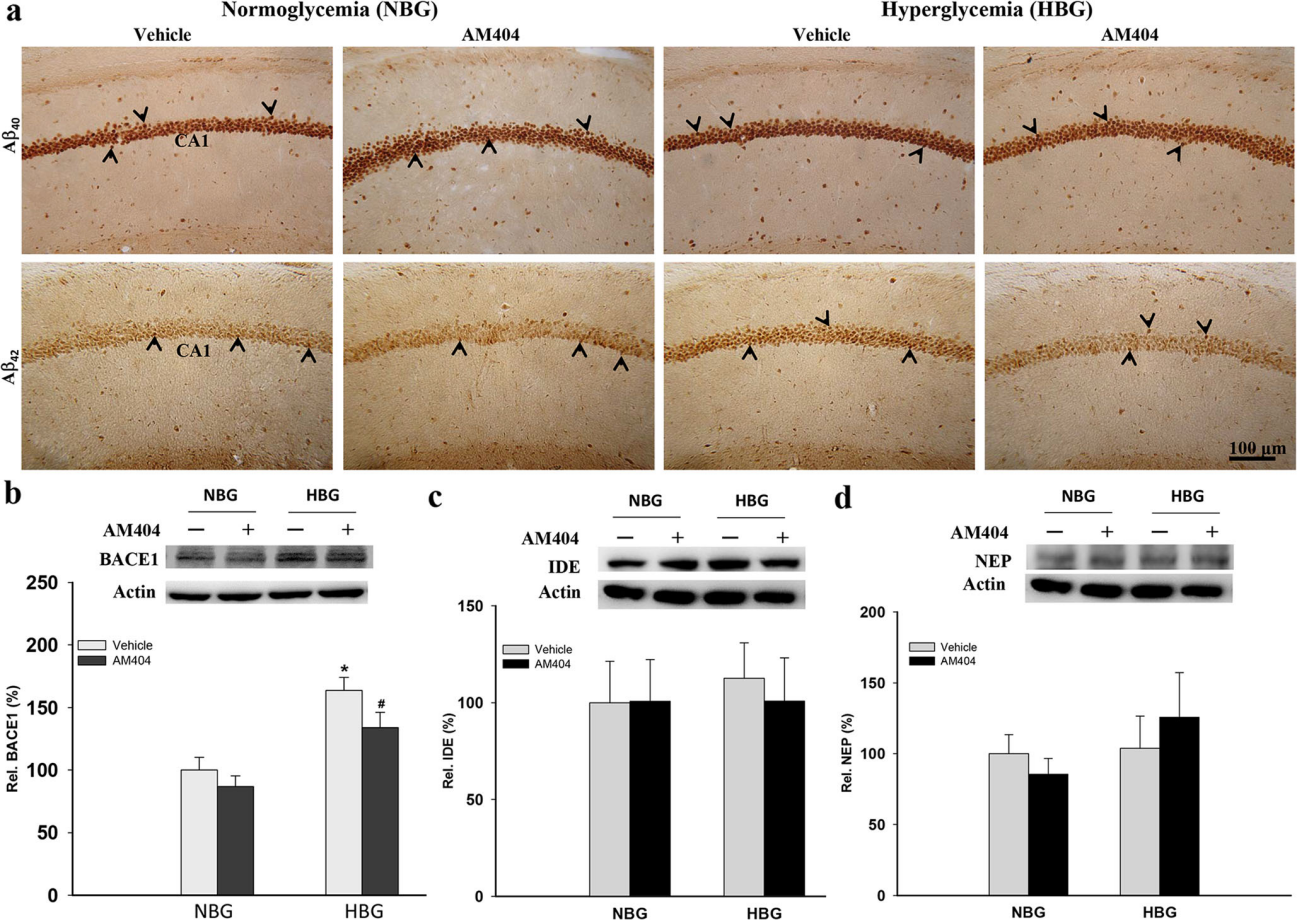
Effect of AM404 on the Aβ levels in the hippocampi of hyperglycemic 3×Tg-AD mice. **a** Representative immunohistochemical staining of amyloid deposits in the mouse hippocampal CA1 region using Aβ_40_ and Aβ_42_ antibodies. Scale bar = 100 μm. The arrowheads indicate the positive staining (*n* = 3–4 per group; the quantitative results are shown in Table 1). Representative western blots and quantitative densitometry results for the BACE1 (**b**), IDE (**c**), and NEP (**d**) expression levels. β-Actin was used as an internal control. The quantitative data are presented as the means ± SEM of each group (*n* = 3–5 per group). Asterisk, compared to the NBG with vehicle group; number sign, compared to the HBG with vehicle group (*^, #^*P* < 0.05)

Intracellular Aβ deposition in the hippocampus plays an important role in the cognitive dysfunction of AD. Therefore, we examined the intracellular Aβ levels in the hippocampi of 3×Tg-AD mice. Significantly increased Aβ_40_ and Aβ_42_ levels were detected in the HBG group compared with the NBG group (*P* < 0.05–0.01; Fig. 3a; Table 1), and the AM404 treatment reduced the Aβ_40_ (*P* < 0.01) and Aβ_42_ (*P* < 0.05) levels compared to the vehicle-treated HBG group (Fig. 3a; Table 1) (*n* = 3–5). Hyperglycemia increased BACE1 expression, which was significantly reduced by AM404 administration (*P* < 0.05; Fig. 3b). In addition, hyperglycemia or AM404 had no effects on IDE and NEP expression (Fig. 3c, d). Therefore, these results indicate that AM404 attenuated Aβ production in the hippocampi of 3×Tg-AD mice under hyperglycemic conditions, which may result from reduced BACE1 expression.

**Table 1:** Quantitative results of the immunohistochemical staining

	Normoglycemia (NBG)		Hyperglycemia (HBG)	
	Vehicle	AM404	Vehicle	AM404
Aβ_40_	172.17 ± 7.61	181.88 ± 4.17	212.92 ± 10.49 **↑↑**^a^	164.78 ± 9.51 **↓↓**^b^
Aβ_42_	27.50 ± 1.20	29.13 ± 0.64	33.00 ± 2.07 **↑**^a^	26.91 ± 1.28 **↓**^b^
Hypertrophic astrocytes (GFAP)	2.50 ± 0.42	3.33 ± 1.02	43.19 ± 3.46 **↑↑↑**^a^	15.63 ± 2.05 **↓↓↓**^b^
Activated microglia (Iba1)	36.08 ± 1.16	34.25 ± 2.19	62.53 ± 1.16 **↑↑↑**^a^	29.43 ± 0.78 **↓↓↓**^b^
Serotonergic neurons (5HT)	35.67 ± 0.92	31.80 ± 2.04	19.00 ± 1.34 **↓↓↓**^a^	33.00 ± 1.78 **↑↑↑**^b^
Noradrenergic neurons (TH)	75.60 ± 4.97	75.75 ± 3.12	45.79 ± 2.66 **↓↓↓**^a^	56.89 ± 4.45 **↑**^b^

Data are shown as the mean ± SEM

^a^Compared with NBG/vehicle

^b^Compared with HBG/vehicle

↑ Increased (*P* < 0.05); ↑↑ increased (*P* < 0.01); ↑↑↑ increased (*P* < 0.001)

↓ Decreased (*P* < 0.05); ↓↓ decreased (*P* < 0.01); ↓↓↓ decreased (*P* < 0.001)

### AM404 suppresses inflammation and reduced the neuronal loss in hyperglycemic 3×Tg-AD mice

**Fig. 4:**
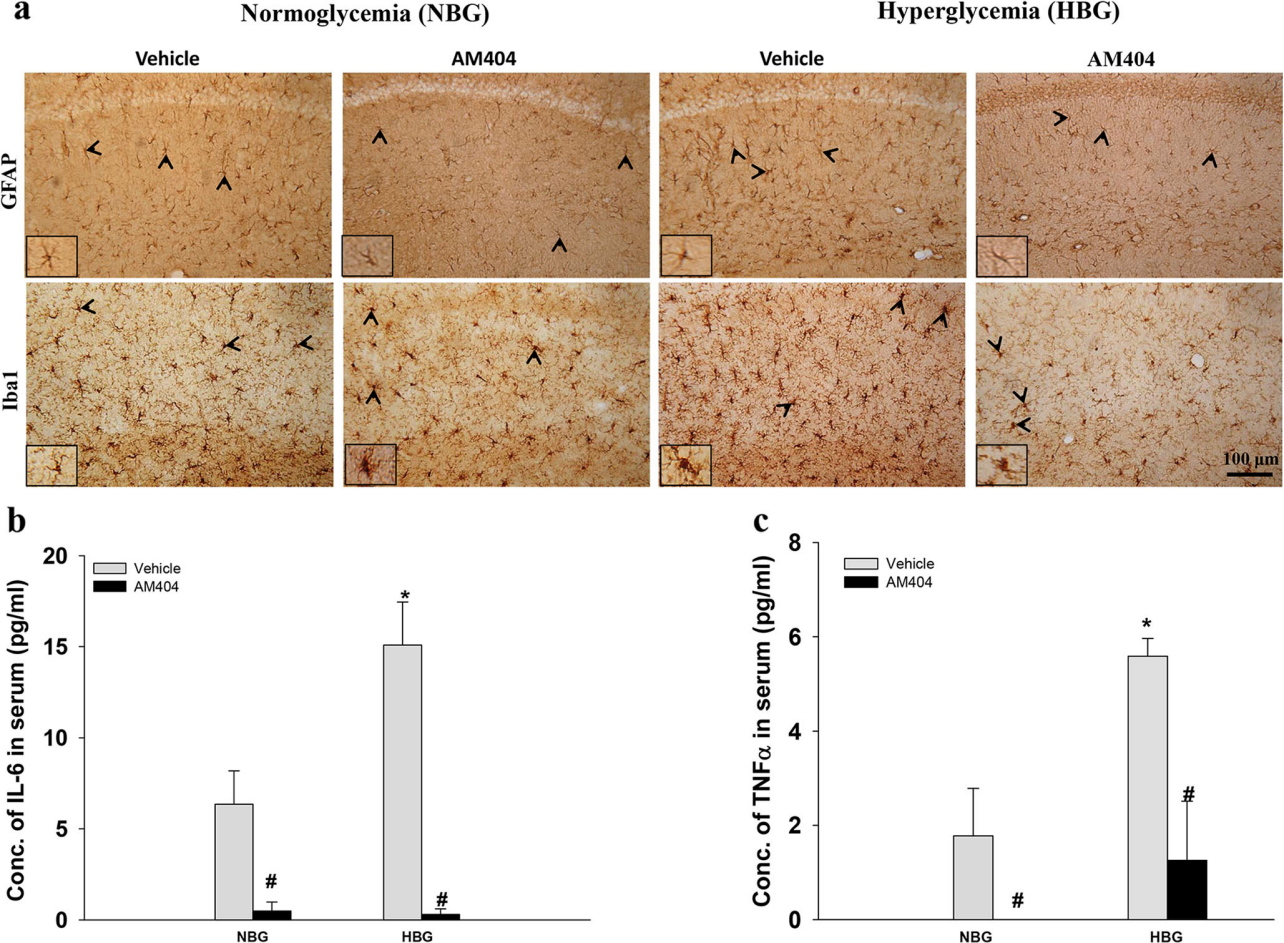
Effect of AM404 on inflammation in hyperglycemic 3×Tg-AD mice. **a** Representative immunohistochemical staining of astrocytes using an anti-GFAP antibody and microglia using an anti-Iba1 antibody in mouse hippocampal tissue. Scale bar = 100 μm. The arrowheads indicate the positive staining for activated microglia and astrocytes, which are magnified in the insets of each figure (*n* = 3–4 per group; the quantitative results are shown in Table 1). **b** Levels of the inflammatory cytokine IL-6 as determined in mouse serum by ELISA (*n* = 3–5 per group). **c** Levels of the inflammatory cytokine TNFα as determined in mouse serum by ELISA (*n* = 3–5 per group). Asterisk, compared to the NBG with vehicle group; number sign, compared to the HBG with vehicle group (*^, #^*P* < 0.05)

Neuroinflammation in the mouse hippocampus was substantially increased by hyperglycemia as determined by GFAP (hypertrophic astrocytes, *P* < 0.001) and Iba1 (activated microglia, *P* < 0.001) staining (Fig. 4a; Table 1). AM404 effectively attenuated both astrogliosis and microgliosis (*P* < 0.001; Fig. 4a; Table 1). Furthermore, the HBG groups showed elevated serum levels of the inflammatory cytokines IL-6 and TNF-α, which were also ameliorated by the AM404 treatment (*P* < 0.05; Fig. 4b, c). In addition, the AM404 treatment significantly reduced the IL-6 and TNF-α levels in the NBG groups (*P* < 0.05; Fig. 4b, c). Furthermore, according to the immunohistochemical analyses, AM404 significantly protected serotonergic (*P* < 0.001) and noradrenergic neurons (*P* < 0.05) in hyperglycemic 3×Tg-AD mice (Table 1; Supporting Information S5). These results revealed that chronic low dose of AM404 has strong anti-inflammatory properties and reduced the loss of serotonergic and noradrenergic neurons in hyperglycemic 3×Tg-AD mice.

## Discussion

Based on the data presented in the current study, administration of AM404 at low dose significantly induced neuroprotection in primary mouse hippocampal neuronal culture incubated with WT/GFX and reduced cognitive deficits associated pathological features, which might be partly through Akt/GSK3β pathway in hyperglycemic 3×Tg-AD mice. Therefore, we propose that chronic low dose of AM404 may be used as an indirect GSK3β inhibitor in AD.

From our in vitro results, WT/GFX application in mouse primary hippocampal neuronal culture reduced the levels of pS9-GSK3β (inactive form) expression and increased tau protein phosphorylation at S396 site. These results were consistent with the previous study (Shi et al. 2008). However, the administration of AM404 at 0.5 μM induced the neuroprotection against the toxicity-induced by WT/GFX in primary hippocampal neuronal culture. In addition, as described in recent investigations (Xiang et al. 2015; Chao et al. 2016), the current study used hyperglycemia to increase spatial learning and memory deficits associated with more severe pathological presentations in 3×Tg-AD mice. Cognitive dysfunction associated with neuropathological hallmarks, such as amyloid deposition, tau protein hyperphosphorylation, and gliosis, was used to highlight several primary concerns during AD and diabetes mellitus (DM) (Halmos and Suba 2016). Previous evidence has also suggested that impaired glucose metabolism in mild cognitive impairment (MCI) is associated with increased progression to AD (Morris et al. 2014). Furthermore, hyperglycemia has been shown to increase the prevalence of AD in APP/PS1 transgenic mice (Wang et al. 2014). Therefore, we confirmed that the induction of hyperglycemia increased the progression of cognitive dysfunction and pathological features in 3×Tg-AD mice. In addition, the chronic systemic administration of AM404 (0.25 mg/kg) improved spatial learning and memory in the hyperglycemic 3×Tg-AD mice. Our previous biochemical assay identified that the concentration of the compound necessary to half-maximally inhibit GSK-3β kinase activity (IC50) is 5.353 μM (Lin et al. 2016), which is much less potent than the known inhibitors, SB216763 (IC50 = 0.018 μM) and SB415286 (IC50 = 0.248 μM). In addition, it has been reported that the dose of AM404 targeting on the TRPV1 and cannabinoid (CB) receptors exceeds 10 μM (in vitro) and 10 mg/kg (in vivo; i.p.) (Pertwee 1997; Saliba et al. 2017). Therefore, the effective doses of AM404 used in this study, 0.5 μM (in vitro) and 0.25 mg/kg (in vivo), might not work as a direct GSK3β inhibitor and independent on TRPV1 and CB1 receptors. In addition to the specific targets CB1 or TRPV1 receptors, the effects of AM404 might be also dependent on nonspecific targets, including anti-oxidative stress through a receptor-independent mechanism (La Rana et al. 2006; Soliman et al. 2016). Therefore, we suspect the positive effects of AM404 in our study might be through nonspecific targets. After carefully examined the results of this study, we suggest Akt could be the target of AM404. Therefore, we proposed a pathway for the current study (Supporting Information S6). As shown in the figure, once the Akt is activated by AM404, the activated Akt (pS473Akt) could phosphorylate GSK3β (at S9 site, and become an inactive form), which could further reduce the features of AD, including Aβ, tau, inflammation, and cognitive impairment. Another study further indicated that the concentration of anandamide in rat plasma was increased in a time-dependent manner after systemic administration of AM404 (10 mg/kg, i.p.) (Giuffrida et al. 2000). Therefore, chronic administration of AM404 at low dose attenuated cognitive deficit and related pathological features associated with Akt/GSK3β pathway, which might be increased by the concentration of anandamide in plasma or brain.

In this study, hyperglycemia increased BACE1 expression, Aβ deposition, tau protein phosphorylation, gliosis, and peripheral inflammation and decreased the levels of activated Akt and pS9-GSK3β (the inactive form of GSK3β) in 3×Tg-AD mice, whereas the AM404 treatment reversed these changes. Extracellular protofibrillar aggregates interfere directly with synaptic efficacy and play a crucial role on learning (Mohandas et al. 2009). However, many evidences also suggest that intraneuronal Aβ accumulation impairs axonal transport and synaptic transmission thereby playing a role in the cognitive impairment associated with AD (Stokin et al. 2005; Ripoli et al. 2014). Intracellular Aβ deposition is the first clear neuropathological manifestation closely associated with synaptic dysfunction in the brains of 3×Tg-AD mice or other AD transgenic mouse models (Kuo et al. 2001; Wirths et al. 2001; Oddo et al. 2003). The intraneuronal Aβ was firstly revealed within neurons at 3 weeks of age in 3xTg-AD mice (Oh et al. 2010); however, extracellular aggregates were not observed until 12–14 months old (Oddo et al. 2003; Yan et al. 2012). Recent evidence showed that high glucose leads to the increased expression of genes related to Aβ production, such as BACE1 (Nagai et al. 2016). The chronic systemic administration of AM404 at low dose reduced intracellular Aβ40 and Aβ42, which may be due to the reduction in BACE1 expression because no alteration was observed in the Aβ-metabolizing enzymes (IDE, NEP). Although we did not examine the enzymatic activity of these three enzymes including BACE1, IDE, and NEP, accumulating evidences have shown that the expression levels of production (BACE1) and degradation (IDE or NEP) enzymes of Aβ were consistently regulated with the enzymatic activities in the AD brains (Fukumoto et al. 2002; Austin et al. 2010; McAllister et al. 2010; Marwarha et al. 2017; Li et al. 2018). Moreover, the reduction in the levels of pS473Akt and pS9-GSK3β in the brains of STZ-treated mice is consistent with the results of a recent study (Chowdhury et al. 2016). In addition, GSK3β activity has been suggested to modulate BACE1 expression and subsequently regulate Aβ_42_ deposition (Zhou et al. 2016). Furthermore, Aβ_25–35_ decreases Akt activation and increases the level of pS9-GSK3β (Naert et al. 2015). Thus, AM404 may inhibit GSK3β activity and subsequently regulate Aβ deposition via an increase of pAkt expression levels to protect against hyperglycemic toxicity in 3×Tg-AD mice. A previous study suggested that the GSK3β phosphorylation sites corresponding to the formation of paired helical tau filaments include Ser202, Ser396, Thr181, and Thr231, according to the longest human tau isoform, which contains 441 residues (Hanger et al. 1998). Recent evidence suggests that Aβ deposition and accumulation of phosphorylated tau proteins lead to inflammation in 3×Tg-AD mice (Ontiveros-Torres et al. 2016). Furthermore, the anti-inflammatory property of AM404 observed here was consistent with a previous study (Roche et al. 2008). Therefore, chronic systemic administration of AM404 at low dose used maybe reverse cognitive deficits via Akt/GSK3β pathway, not dependent on a direct GSK3β inhibitor, CB1, or TRP1 receptor in hyperglycemic 3×Tg-AD mice.

In summary, our results show that chronic systemic low dose of AM404 may serve as a potential therapy for AD.

## Electronic supplementary material

ESM 1 (DOCX 1398 kb)

## References

[CR1] Abush H, Akirav I (2010). Cannabinoids modulate hippocampal memory and plasticity. Hippocampus.

[CR2] Adamczyk P, Golda A, McCreary AC, Filip M, Przegalinski E (2008). Activation of endocannabinoid transmission induces antidepressant-like effects in rats. J Physiol Pharmacol.

[CR3] Austin SA, Santhanam AV, Katusic ZS (2010). Endothelial nitric oxide modulates expression and processing of amyloid precursor protein. Circ Res.

[CR4] Barna I, Soproni K, Arszovszki A, Csabai K, Haller J (2007). WIN-55,212-2 chronically implanted into the CA3 region of the dorsal hippocampus impairs learning: a novel method for studying chronic, brain-area-specific effects of cannabinoids. Behav Pharmacol.

[CR5] Bitencourt RM, Pamplona FA, Takahashi RN (2008). Facilitation of contextual fear memory extinction and anti-anxiogenic effects of AM404 and cannabidiol in conditioned rats. Eur Neuropsychopharmacol.

[CR6] Braak H, Braak E (1991). Demonstration of amyloid deposits and neurofibrillary changes in whole brain sections. Brain Pathol.

[CR7] Braak H, Braak E (1991). Neuropathological stageing of Alzheimer-related changes. Acta Neuropathol.

[CR8] Campolongo P, Trezza V, Ratano P, Palmery M, Cuomo V (2011). Developmental consequences of perinatal cannabis exposure: behavioral and neuroendocrine effects in adult rodents. Psychopharmacology.

[CR9] Chao AC, Lee TC, Juo SH, Yang DI (2016). Hyperglycemia increases the production of amyloid beta-peptide leading to decreased endothelial tight junction. CNS Neurosci Ther.

[CR10] Chowdhury S, Ghosh S, Rashid K, Sil PC (2016). Deciphering the role of ferulic acid against streptozotocin-induced cellular stress in the cardiac tissue of diabetic rats. Food Chem Toxicol.

[CR11] D’Angelo B, Ek CJ, Sun Y (2016). GSK3beta inhibition protects the immature brain from hypoxic-ischaemic insult via reduced STAT3 signalling. Neuropharmacology.

[CR12] Dolan PJ, Johnson GV (2010). The role of tau kinases in Alzheimer’s disease. Curr Opin Drug Discov Devel.

[CR13] Ferri CP, Prince M, Brayne C, Brodaty H, Fratiglioni L, Ganguli M, Hall K, Hasegawa K, Hendrie H, Huang Y, Jorm A, Mathers C, Menezes PR, Rimmer E, Scazufca M, Alzheimer’s Disease International (2005). Global prevalence of dementia: a Delphi consensus study. Lancet.

[CR14] Fukumoto H, Cheung BS, Hyman BT, Irizarry MC (2002). Beta-secretase protein and activity are increased in the neocortex in Alzheimer disease. Arch Neurol.

[CR15] Garcia-Arencibia M, Gonzalez S, de Lago E (2007). Evaluation of the neuroprotective effect of cannabinoids in a rat model of Parkinson’s disease: importance of antioxidant and cannabinoid receptor-independent properties. Brain Res.

[CR16] Giuffrida A, Rodriguez de Fonseca F, Nava F, Loubet-Lescoulie P, Piomelli D (2000) Elevated circulating levels of anandamide after administration of the transport inhibitor, AM404, Eur J Pharmacol 408(2): 161–168. DOI: S0014–2999(00)00786-X. 10.1016/s0014-2999(00)00786-x 11080522

[CR17] Guo C, Zhang S, Li JY, Ding C, Yang ZH, Chai R, Wang X, Wang ZY (2016). Chronic hyperglycemia induced via the heterozygous knockout of Pdx1 worsens neuropathological lesion in an Alzheimer mouse model. Sci Rep.

[CR18] Halmos T, Suba I (2016). Alzheimer’s disease and diabetes—the common pathogenesis. Neuropsychopharmacol Hung.

[CR19] Hanger DP, Betts JC, Loviny TL, Blackstock WP, Anderton BH (1998). New phosphorylation sites identified in hyperphosphorylated tau (paired helical filament-tau) from Alzheimer’s disease brain using nanoelectrospray mass spectrometry. J Neurochem.

[CR20] Hill MN, McLaughlin RJ, Bingham B (2010). Endogenous cannabinoid signaling is essential for stress adaptation. Proc Natl Acad Sci U S A.

[CR21] Hogestatt ED, Jonsson BA, Ermund A (2005). Conversion of acetaminophen to the bioactive N-acylphenolamine AM404 via fatty acid amide hydrolase-dependent arachidonic acid conjugation in the nervous system. J Biol Chem.

[CR22] Huang HJ, Chen SL, Hsieh-Li HM (2015). Administration of NaHS attenuates footshock-induced pathologies and emotional and cognitive dysfunction in triple transgenic Alzheimer’s mice. Front Behav Neurosci.

[CR23] Huang HJ, Chen YH, Liang KC, Jheng YS, Jhao JJ, Su MT, Lee-Chen GJ, Hsieh-Li HM (2012). Exendin-4 protected against cognitive dysfunction in hyperglycemic mice receiving an intrahippocampal lipopolysaccharide injection. PLoS One.

[CR24] Kuo YM, Beach TG, Sue LI, Scott S, Layne KJ, Kokjohn TA, Kalback WM, Luehrs DC, Vishnivetskaya TA, Abramowski D, Sturchler-Pierrat C, Staufenbiel M, Weller RO, Roher AE (2001). The evolution of A beta peptide burden in the APP23 transgenic mice: implications for A beta deposition in Alzheimer disease. Mol Med.

[CR25] La Rana G, Russo R, Campolongo P, Bortolato M, Mangieri RA, Cuomo V, Iacono A, Raso GM, Meli R, Piomelli D, Calignano A (2006) Modulation of neuropathic and inflammatory pain by the endocannabinoid transport inhibitor AM404 [N-(4-hydroxyphenyl)-eicosa-5,8,11,14-tetraenamide]. J Pharmacol Exp Ther 317:1365–1371. 10.1124/jpet.105.100792 10.1124/jpet.105.100792 16510698

[CR26] Li H, Wu J, Zhu L, Sha L, Yang S, Wei J, Ji L, Tang X, Mao K, Cao L, Wei N, Xie W, Yang Z (2018). Insulin degrading enzyme contributes to the pathology in a mixed model of type 2 diabetes and Alzheimer’s disease: possible mechanisms of IDE in T2D and AD. Biosci Rep.

[CR27] Lin CH, Hsieh YS, Wu YR, Hsu CJ, Chen HC, Huang WH, Chang KH, Hsieh-Li HM, Su MT, Sun YC, Lee GC, Lee-Chen GJ (2016). Identifying GSK-3beta kinase inhibitors of Alzheimer’s disease: virtual screening, enzyme, and cell assays. Eur J Pharm Sci.

[CR28] Lovestone S, Boada M, Dubois B (2015). A phase II trial of tideglusib in Alzheimer’s disease. J Alzheimers Dis.

[CR29] Marwarha G, Rostad S, Lilek J, Kleinjan M, Schommer J, Ghribi O (2017). Palmitate increases beta-site AbetaPP-cleavage enzyme 1 activity and amyloid-beta genesis by evoking endoplasmic reticulum stress and subsequent C/EBP homologous protein activation. J Alzheimers Dis.

[CR30] McAllister C, Long J, Bowers A, Walker A, Cao P, Honda SI, Harada N, Staufenbiel M, Shen Y, Li R (2010). Genetic targeting aromatase in male amyloid precursor protein transgenic mice down-regulates beta-secretase (BACE1) and prevents Alzheimer-like pathology and cognitive impairment. J Neurosci.

[CR31] Miller JS, Barr JL, Harper LJ, Poole RL, Gould TJ, Unterwald EM (2014). The GSK3 signaling pathway is activated by cocaine and is critical for cocaine conditioned reward in mice. PLoS One.

[CR32] Mohandas E, Rajmohan V, Raghunath B (2009). Neurobiology of Alzheimer’s disease. Indian J Psychiatry.

[CR33] Morris JK, Vidoni ED, Honea RA, Burns JM (2014). Impaired glycemia increases disease progression in mild cognitive impairment. Neurobiol Aging.

[CR34] Naert G, Ferre V, Meunier J (2015). Leucettine L41, a DYRK1A-preferential DYRKs/CLKs inhibitor, prevents memory impairments and neurotoxicity induced by oligomeric Abeta25-35 peptide administration in mice. Eur Neuropsychopharmacol.

[CR35] Nagai N, Ito Y, Sasaki H (2016). Hyperglycemia enhances the production of amyloid beta1-42 in the lenses of Otsuka Long-Evans Tokushima fatty rats, a model of human type 2 diabetes. Invest Ophthalmol Vis Sci.

[CR36] Oddo S, Caccamo A, Shepherd JD, Murphy MP, Golde TE, Kayed R, Metherate R, Mattson MP, Akbari Y, LaFerla FM (2003). Triple-transgenic model of Alzheimer’s disease with plaques and tangles: intracellular Abeta and synaptic dysfunction. Neuron.

[CR37] Oh KJ, Perez SE, Lagalwar S, Vana L, Binder L, Mufson EJ (2010). Staging of Alzheimer’s pathology in triple transgenic mice: a light and electron microscopic analysis. Int J Alzheimers Dis.

[CR38] Ontiveros-Torres MA, Labra-Barrios ML, Diaz-Cintra S (2016). Fibrillar amyloid-beta accumulation triggers an inflammatory mechanism leading to hyperphosphorylation of the carboxyl-terminal end of tau polypeptide in the hippocampal formation of the 3xTg-AD transgenic mouse. J Alzheimers Dis.

[CR39] Patel S, Hillard CJ (2006). Pharmacological evaluation of cannabinoid receptor ligands in a mouse model of anxiety: further evidence for an anxiolytic role for endogenous cannabinoid signaling. J Pharmacol Exp Ther.

[CR40] Patel S, Roelke CT, Rademacher DJ, Cullinan WE, Hillard CJ (2004). Endocannabinoid signaling negatively modulates stress-induced activation of the hypothalamic-pituitary-adrenal axis. Endocrinology.

[CR41] Pertwee RG (1997) Pharmacology of cannabinoid CB1 and CB2 receptors. Pharmacol Ther 74(2):129–180 10.1016/s0163-7258(97)82001-3 9336020

[CR42] Querfurth HW, LaFerla FM (2010). Alzheimer’s disease. N Engl J Med.

[CR43] Ripoli C, Cocco S, Li Puma DD, Piacentini R, Mastrodonato A, Scala F, Puzzo D, D’Ascenzo M, Grassi C (2014). Intracellular accumulation of amyloid-beta (Abeta) protein plays a major role in Abeta-induced alterations of glutamatergic synaptic transmission and plasticity. J Neurosci.

[CR44] Roche M, Kelly JP, O’Driscoll M, Finn DP (2008). Augmentation of endogenous cannabinoid tone modulates lipopolysaccharide-induced alterations in circulating cytokine levels in rats. Immunology.

[CR45] Saliba SW, Marcotegui AR, Fortwangler E, Ditrich J, Perazzo JC, Munoz E, de Oliveira ACP, Fiebich BL (2017) AM404, paracetamol metabolite, prevents prostaglandin synthesis in activated microglia by inhibiting COX activity. J Neuroinflammation 14:246. 10.1186/s12974-017-1014-3 10.1186/s12974-017-1014-3 PMC5729401 29237478

[CR46] Shi HR, Zhu LQ, Wang SH, Liu XA, Tian Q, Zhang Q, Wang Q, Wang JZ (2008). 17beta-estradiol attenuates glycogen synthase kinase-3beta activation and tau hyperphosphorylation in Akt-independent manner. J Neural Transm (Vienna).

[CR47] Soliman E, Van Dross R (2016) Anandamide-induced endoplasmic reticulum stress and apoptosis are mediated by oxidative stress in non-melanoma skin cancer: receptor-independent endocannabinoid signaling. Mol Carcinog 55:1807–1821. 10.1002/mc.22429 10.1002/mc.22429 26513129

[CR48] Stokin GB, Lillo C, Falzone TL, Brusch RG, Rockenstein E, Mount SL, Raman R, Davies P, Masliah E, Williams DS, Goldstein LS (2005). Axonopathy and transport deficits early in the pathogenesis of Alzheimer’s disease. Science.

[CR49] Wang X, Yu S, Hu JP, Wang CY, Wang Y, Liu HX, Liu YL (2014). Streptozotocin-induced diabetes increases amyloid plaque deposition in AD transgenic mice through modulating AGEs/RAGE/NF-kappaB pathway. Int J Neurosci.

[CR50] Wirths O, Multhaup G, Czech C et al (2001) Intraneuronal Abeta accumulation precedes plaque formation in beta-amyloid precursor protein and presenilin-1 double-transgenic mice. Neurosci Lett 306(1–2): 116–120. DOI: S0304–3940(01)01876–6. 10.1016/s0304-3940(01)01876-6 11403971

[CR51] Xiang Q, Zhang J, Li CY, Wang Y, Zeng MJ, Cai ZX, Tian RB, Jia W, Li XH (2015). Insulin resistance-induced hyperglycemia decreased the activation of Akt/CREB in hippocampus neurons: molecular evidence for mechanism of diabetes-induced cognitive dysfunction. Neuropeptides.

[CR52] Yan XX, Cai Y, Shelton J, Deng SH, Luo XG, Oddo S, LaFerla FM, Cai H, Rose GM, Patrylo PR (2012). Chronic temporal lobe epilepsy is associated with enhanced Alzheimer-like neuropathology in 3xTg-AD mice. PLoS One.

[CR53] Zani A, Braida D, Capurro V, Sala M (2007). Delta9-tetrahydrocannabinol (THC) and AM 404 protect against cerebral ischaemia in gerbils through a mechanism involving cannabinoid and opioid receptors. Br J Pharmacol.

[CR54] Zhang X, Guo Y, Zhang L, Wen T, Piao Z, Shi H, Chen D, Duan Z, Ren F (2015). Oxidative stress promotes hepatocyte apoptosis mediated by glycogen synthase kinase 3beta. Xi Bao Yu Fen Zi Mian Yi Xue Za Zhi.

[CR55] Zhang Y, Huang NQ, Yan F, Jin H, Zhou SY, Shi JS, Jin F (2018). Diabetes mellitus and Alzheimer’s disease: GSK-3beta as a potential link. Behav Brain Res.

[CR56] Zhou D, Liu H, Li C, Wang F, Shi Y, Liu L, Zhao X, Liu A, Zhang J, Wang C, Chen Z (2016). Atorvastatin ameliorates cognitive impairment, Abeta1-42 production and Tau hyperphosphorylation in APP/PS1 transgenic mice. Metab Brain Dis.

