# Supplementary material for: Chronic low dose of AM404 ameliorates the cognitive impairment and pathological features in hyperglycemic 3xTg-AD mice
Source: Psychopharmacology (Berl). 2018 Nov 13;236(2):763–73. doi: 10.1007/s00213-018-5108-0 (PMC6469654; doi:10.1007/s00213-018-5108-0)
Supplement: Supplementary file 1 — (DOCX 1398 kb) [file 213_2018_5108_MOESM1_ESM.docx]

**Chronic low dose of AM404 ameliorates the cognitive impairment and pathological features in hyperglycemic 3xTg-AD mice**

Hei-Jen Huang^1^, Shu‐Ling Chen^2^, Hsin-Yu Huang^2^, Ying-Chieh Sun^3^, Guan-Chiun Lee^2,4^, Guey-Jen Lee-Chen^2^, Hsiu Mei Hsieh-Li^2,^*, and Ming-Tsan Su^2,^**

^1^Department of Nursing, Mackay Junior College of Medicine, Nursing and Management, Taipei 11260, Taiwan

^2^Department of Life Science, ^3^Department of Chemistry, National Taiwan Normal University, Taipei 11677, Taiwan

^4^MSSBIO CO., LTD., Hsin-chu 30072, Taiwan

**Running Title**: AM404 attenuates the impairment in AD.

*Correspondence

H. M. Hsieh-Li, Ph.D., Professor, Department of Life Science, National Taiwan Normal University, Taipei, PO Box 11677, Taiwan

Tel.: +886 2 77346354

Fax: +886 2 29312904

E-mail: [hmhsieh@ntnu.edu.tw](mailto:hmhsieh@ntnu.edu.tw)

** Correspondence

M. T. Su, Ph.D., Associate professor, Department of Life Science, National Taiwan Normal University, Taipei, PO Box 11677, Taiwan

Tel.: +886 2 77346354

Fax: +886 2 29312904

E-mail: mtsu@ntnu.edu.tw

**Supplementary Methods**

**Murine primary hippocampal neuron cultures and compound treatment**

We used pregnant female C57BL/6J mice to establish the murine primary neuronal cultures. Hippocampi were isolated from embryos (days 16-18). The isolated tissues were trypsinized (0.05%) for 15 min at 37°C, and cells were cultured using previously described methods ([Huang et al. 2015](#_ENREF_1)). On days *in vitro* (DIV) 4 and 7, cytosine arabinoside (2 μM) was added to the mouse hippocampal cultures to reduce the glial cell populations. On DIV 9, the cells were treated with 10 nM Wortmannin (WT) and GF109203X (GFX) to induce tau hyperphosphorylation to mimic the conditions of AD ([Li et al. 2006](#_ENREF_2)). The potential GSK3β inhibitor AM404 (0.1, 0.25, and 0.5 μM) was applied to the primary hippocampal neuronal cultures immediately after the addition of WT/GFX. The cells were harvested for immunocytochemical (ICC) staining (AM404; 0.5 μM) analyses 12 h after treatment (n = 3–5 cultures (3 wells/culture) per group).

**Immunocytochemical (ICC) staining**

For ICC staining, harvested cells (n = 3–5 cultures (3 wells/culture) per group) were fixed with ice-cold 4% paraformaldehyde (PFA; Sigma, St. Louis, MO, USA) for 30 min and washed with phosphate-buffered saline (PBS) containing Triton X-100 (PBST) 3 times for 10 min each. Nonspecific reactivity was blocked by incubating cells with 10% fetal bovine serum (FBS) for 2 h. Cells were then incubated with primary antibodies against NeuN (1:1000; Millipore, Temecula, CA, USA) and MAP2 (1:1000; Millipore, Temecula, CA, USA) for 16 hr at 4°C, followed by an incubation with a secondary antibody for 2 h at 37°C. Finally, the nuclei of all neuronal cells were counterstained with 4’,6-diamino-2-phenylindole (DAPI; Sigma, St. Louis, MO, USA) and immediately analyzed using a high content micro-imaging acquisition and screening system (Molecular Devices; Sunnyvale, CA, USA). Both the percentages of neurons and the neurite lengths were analyzed using MetaXpress application software (Molecular Devices).

**Immunoblot analysis**

Cultured neuronal cells (n = 3–5 cultures (3 wells/culture) per group) were homogenized, and the concentrations of the isolated proteins were determined using a bicinchoninic acid (BCA) protein assay kit (Thermo Fisher Scientific, Rockford, IL, USA). The homogenates (25 μg of protein each) were subsequently separated using SDS-PAGE and transferred to polyvinylidene fluoride (PVDF) membranes. Blots were then blocked with 5% (w/v) skim milk to reduce nonspecific binding and probed with the following primary antibodies: pS9-GSK3β (1:1000; Cell Signaling Technology, Danvers, MA, USA), GSK3β (1:1000; Cell Signaling Technology, Danvers, MA, USA), the anti-phospho-tau antibodies S396 (1:1000; Invitrogen, Rockford, IL, USA), and β-actin (1:2000; Millipore, Temecula, CA, USA). These incubations were followed by incubations with horseradish peroxidase (HRP)-conjugated anti-mouse or anti-rabbit IgG (1:10,000; Amersham Pharmacia Biotech; Piscataway, NJ, USA). Protein bands were scanned using an enhanced chemiluminescence detection system (Amersham Pharmacia Biotech; Piscataway, NJ, USA). The immunoreactive bands were quantified using an LAS-4000 chemiluminescence detection system (Fujifilm; Tokyo, Japan).

***Drosophila* tauopathy model and compound treatment**

Eq-Gal4 and UAS-tau fly lines were kindly provided by Drs. H. Sun and M. Feany, respectively ([Wittmann et al. 2001](#_ENREF_49); [Tang et al. 2002](#_ENREF_46)). A tauopathy model generated by overexpressing tau in the nota of *Drosophila* (Eq > tau) was created by meiotic recombination according to previously described methods ([Yeh et al. 2010](#_ENREF_52)). To administer AM404, Eq > tau flies were fed 25 µM or 50 µM of AM404 that had been dissolved in DMSO and mixed with standard cornmeal medium. Control flies raised in the absence of AM404 were fed cornmeal medium with 0.1% DMSO alone. The flies were raised at 25°C under a 12-h light:12-h dark cycle.

**Bristle quantification in Eq > tau flies**

To quantify notal bristles, one-day-old male flies were frozen and immobilized on glass slides using polyvinyl acetate resin adhesive (Nanpao; Taiwan) with their dorsal sides facing upward. Serial focal plane images of notum bristles were recorded using a microscope (Leitz Orthoplan) fitted with a digital camera (CoolSnap 5.0; Photometrics). Serial images with different focal planes were focused using Helicon focus software (Heliconsoft). The numbers of bristles were scored manually.

**Supplementary Results**

**The WT/GFX treatment induces tau toxicity in primary mouse hippocampal neuron cultures**

After a 12-h incubation with WT/GFX, cells were harvested to examine the tau toxicity in primary cultures. The WT/GFX treatment significantly decreased the inactivated pS9-GSK3β/GSK3β ratio (P < 0.001; Figure S1a and b) and increased the levels of the phospho-tau protein (Ser 396) (P < 0.001; Figure S1a and c) in the primary hippocampal neuronal cultures.

**AM404 induces neuroprotection in primary mouse hippocampal neuron cultures**

We applied three different doses (0.1, 0.25, and 0.5 μM) of AM404 to primary mouse hippocampal neuron cultures under WT/GFX-induced toxicity at DIV 9 to evaluate the effects of AM404 on primary cultures. Cells were harvested 12 h later for ICC staining. The neuron numbers and neurite outgrowth measured in the cells are shown in Figure S2. The WT/GFX treatment significantly reduced the neuron numbers (*P* < 0.01; Figure S2a and b) and the average neurite length (*P* < 0.001; Figure S2a and c). However, AM404 exerted significant protective effects on neuronal survival (*P* < 0.05; Figure S2a and b) and neurite length (*P* < 0.05; Figure S2a and c) at both 0.25 and 0.5 μM doses.

**AM404 ameliorates tau toxicity in a *Drosophila* tauopathy model**

As shown in Figure S3, the Eq-gal4 control flies possessed approximately 200 notal bristles each. Treating the Eq-gal4 flies with DMSO did not affect the growth of their notal bristles. In contrast, when treated with DMSO, transgenic flies overexpressing tau driven by Eq-gal4 (Eq > tau) showed dramatically reduced notal bristle numbers. The administration of AM404 at a final concentration of 25 µM or 50 µM reduced the bristle loss phenotype in the Eq > tau flies (*P* < 0.01), indicating that AM404 can ameliorate tau toxicity in *Drosophila*.

**AM404 has no effects on body weight and blood glucose levels in hyperglycemic 3×Tg-AD mice**

Hyperglycemia was induced 3×Tg-AD mice using STZ injections to accelerate AD progression, as previously described ([Wang et al. 2014](#_ENREF_3); [Wang et al. 2015](#_ENREF_4)). As depicted in Figure S4a, STZ (100 mg/kg) was applied to 6-month-old 3×Tg-AD mice on days 1, 2, 8 and 9. AM404 (2.5 mg/kg/day) was then applied to a subgroup of mice via i.p. injection for 4 weeks (Figure S4a). Body weight and blood glucose levels were monitored in the mice throughout the experimental procedure (Figure S4b and c). After the STZ injection, body weights were substantially reduced on days 28 and 43 (*P* < 0.05, Figure S4b). Furthermore, STZ also increased the blood glucose levels 14 days after the STZ injection (*P* < 0.001, Figure S4c). However, no differences in body weights and blood glucose levels were observed between the groups treated with AM404 and vehicle (Figure S4b and c).

**AM404 protects serotonergic and noradrenergic neurons in hyperglycemic 3×Tg-AD mice**

We further examined whether hyperglycemia and AM404 affected neurons related to cognition, including the cholinergic neurons in the medial septum (MS), the vertical diagonal band of Broca (VDB), and the horizontal diagonal band of Broca (HDB) regions; the serotonergic neurons in the raphe nucleus; and the noradrenergic neurons in the locus coeruleus (LC) region. No differences were identified in the cholinergic neurons in the MS/DB regions (data not shown). However, the numbers of both serotonergic neurons in the raphe nucleus and noradrenergic neurons in the LC were significantly reduced in the HBG group (*P* < 0.001, Figure S5a-c). The administration of AM404 effectively protected against the degeneration of the serotonergic neurons in the raphe nucleus (*P* < 0.001; Figure S5a and b) and the noradrenergic neurons in the LC (*P* < 0.05; Figure S5a and c) in the HBG group (n = 3-5). Thus, AM404 exerts neuroprotective effects on a mouse model of AD.

**Supplementary figures**

**
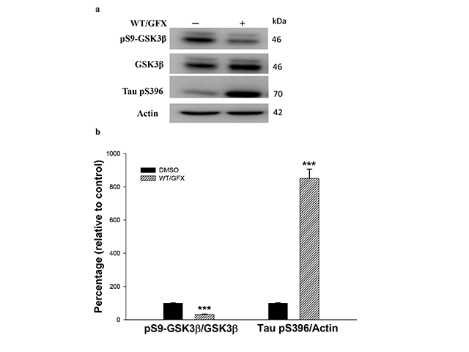
**

**Figure S1. Tau toxicity induced by the WT/GFX treatment in primary murine hippocampal neuron cultures.** (a) Western blot analysis of the expression levels of pS9-GSK3β, p-tau (Ser396) and β-actin, the internal control, in primary cultures treated with WT/GFX. (b) Quantitative analysis of Western blots. ***, *P* < 0.001 compared to the DMSO group. n = 3–5 cultures (3 wells/culture)/group.

**
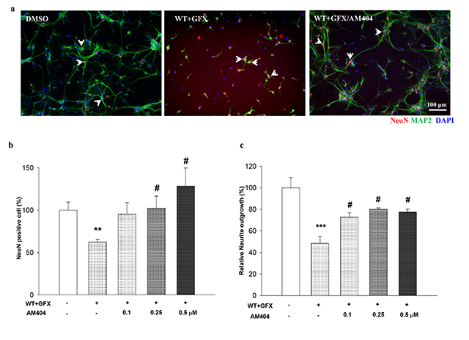
**

**Figure S2. Effect of AM404 on primary neuronal morphology under tau toxicity conditions.** (a) ICC staining of primary cultures using NeuN and MAP2 antibodies. (b-c) Quantitation of NeuN-positive cells (neuronal cell survival) and neurite outgrowth (neurite morphology stained using a MAP2 antibody). **, *P* < 0.01; ***, *P* < 0.001 compared to the control group; #, *P* < 0.05 compared to the WT/GFX group. n = 3–5 cultures (3 wells/culture)/group.


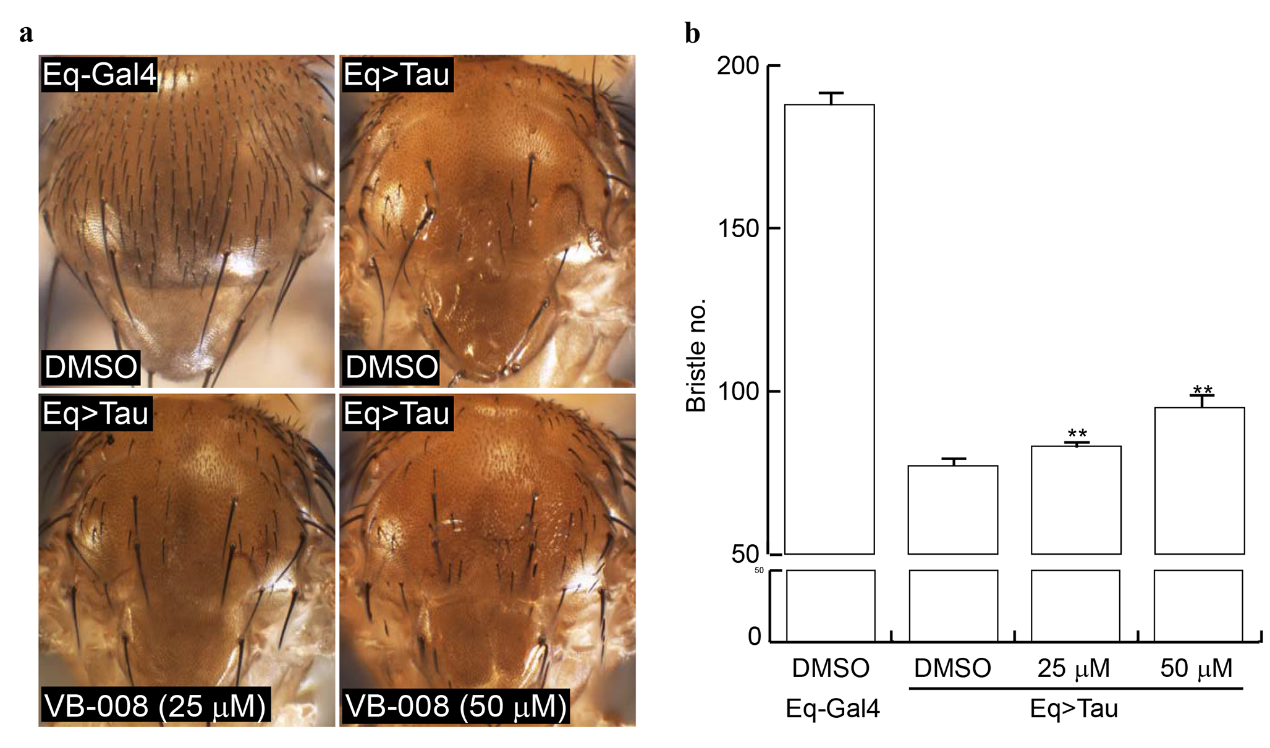


**Figure S3. Effect of AM404 on suppressing tau toxicity in *Drosophila*.** (a) Notal bristle growth was not affected in the Eq-gal4 control flies treated with DMSO. The overexpression of tau driven by Eq-gal4 significantly reduced the number of notal bristles. The administration of AM404 effectively rescued the bristle loss phenotype. (b) For the quantitative analysis, 10 notal bristles were scored from 10 independent animals of each genotype (**, *P* < 0.01).


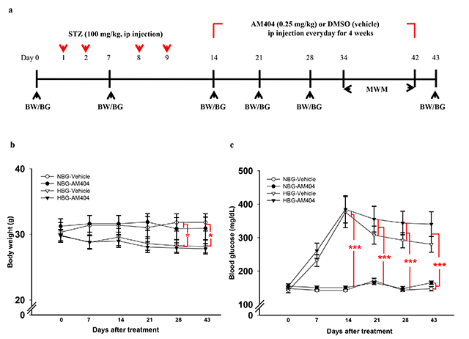


**Figure S4. Effects of AM404 on body weights and blood glucose levels in hyperglycemic 3×Tg-AD mice.** (a) Timeline of the animal treatment protocol used in this study. (b) Body weights measured during the experimental procedure. (c) Blood glucose levels measured during the experimental procedure. *, *P* < 0.05; ***, *P* < 0.001. n = 15 (NBG with vehicle group), 15 (NBG with AM404 group), 8 (HBG with vehicle group), and 9 (HBG with AM404 group).


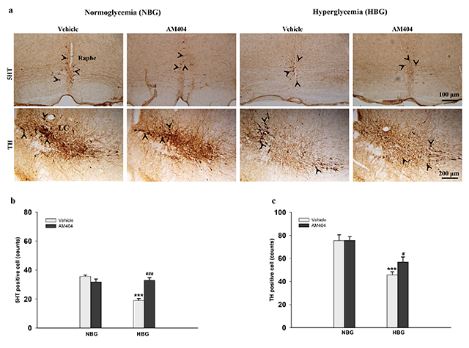


**Figure S5. Neuroprotective effect of AM404 on hyperglycemic 3×Tg-AD mice.** (a) Representative immunohistochemical staining of serotonergic neurons in the raphe nucleus (scale bar = 100 μm) and noradrenergic neurons in the locus coeruleus (LC) (scale bar = 200 μm). The arrowheads indicate positive staining (n=3-4 per group). The quantitative results are shown in (b) and (c), respectively. ***, *P* < 0.001 compared to the NBG with vehicle group; #, *P* < 0.05; ###, *P* < 0.001 compared to the HBG with vehicle group.


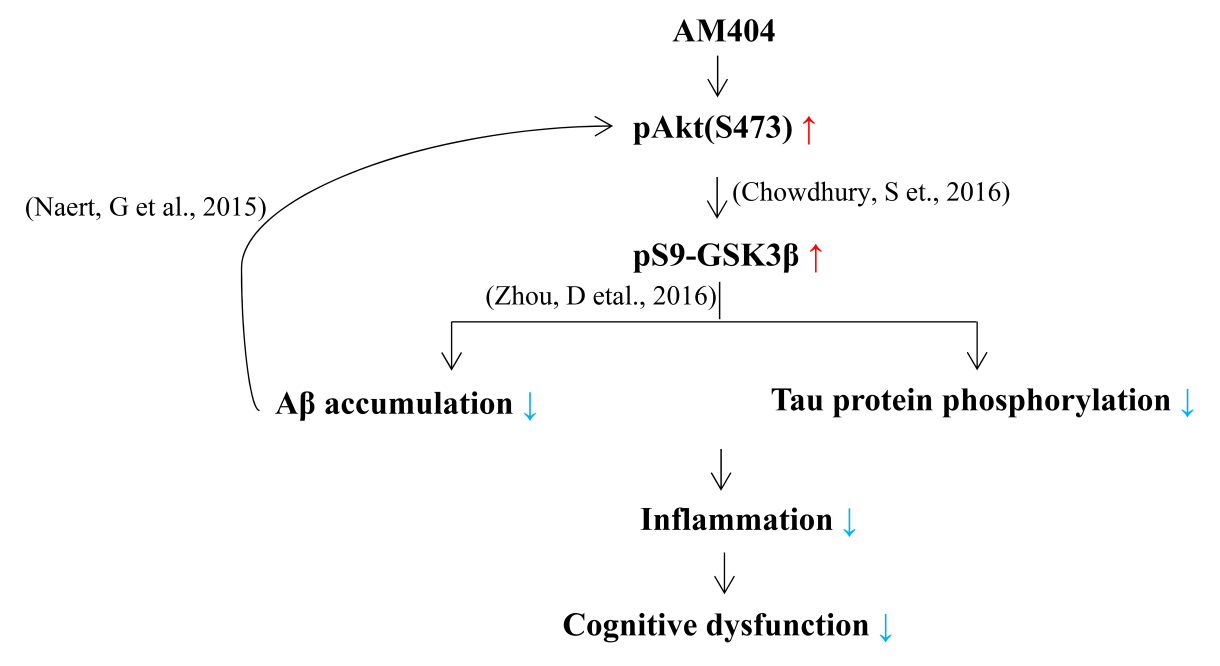


**Figure S6. The hypothesized pathway of low dose AM404 administration in mice**

**References**

Huang HJ, Chen SL, et al. (2015) Administration of NaHS Attenuates Footshock-Induced Pathologies and Emotional and Cognitive Dysfunction in Triple Transgenic Alzheimer's Mice. Front Behav Neurosci 9:312 doi:10.3389/fnbeh.2015.00312

Li X, Lu F, et al. (2006) Activation of glycogen synthase kinase-3 induces Alzheimer-like tau hyperphosphorylation in rat hippocampus slices in culture. J Neural Transm (Vienna) 113:93-102 doi:10.1007/s00702-005-0303-7

Wang X, Yu S, et al. (2014) Streptozotocin-induced diabetes increases amyloid plaque deposition in AD transgenic mice through modulating AGEs/RAGE/NF-kappaB pathway. Int J Neurosci 124:601-608 doi:10.3109/00207454.2013.866110

Wang Y, Wu L, et al. (2015) Synergistic exacerbation of mitochondrial and synaptic dysfunction and resultant learning and memory deficit in a mouse model of diabetic Alzheimer's disease. J Alzheimers Dis 43:451-463 doi:10.3233/JAD-140972
